# An e-Coaching Intervention for Family Carers to Enhance Well-Being and Resilience Through Self-Help Strategies: Protocol for a Randomized Controlled Trial

**DOI:** 10.2196/75944

**Published:** 2026-05-27

**Authors:** Tom Chun Wai Tsoi, Kenneth Yu Hon Kwok, Wai Sze Chan, Vera Mun Yu Tang, Tarani Chandola, Jianchao Quan, Chun Yu Yu, Vivian Weiqun Lou

**Affiliations:** 1 Sau Po Centre on Ageing, Faculty of Social Sciences, The University of Hong Kong, 11/F, T.T. Tsui Building, Pokfulam Road, Hong Kong, 000000, China (Hong Kong); 2 Department of Psychology, Faculty of Social Sciences, University of Hong Kong, Hong Kong, China (Hong Kong); 3 Department of Sociology, Faculty of Social Sciences, University of Hong Kong, Hong Kong, China (Hong Kong); 4 School of Public Health, Li Ka Shing Faculty of Medicine, University of Hong Kong, Hong Kong, China (Hong Kong); 5 Department of Social Work and Social Administration, Faculty of Social Sciences, University of Hong Kong, RM522, The Jockey Club Tower, The Centennial Campus, Pokfulam, Hong Kong, China (Hong Kong), 852 39174835

**Keywords:** online intervention, caregivers, anxiety, depression, 7-Item Generalized Anxiety Disorder Scale, GAD-7, 9-Item Patient Health Questionnaire, PHQ-9, mental health

## Abstract

**Background:**

Family carers of older adults often experience significant mental health challenges, including anxiety and depression. Although online coaching interventions have been found to reduce anxiety and depressive symptoms in carers, only a few studies have examined the broader impact of applying online self-help interventions for enhancing resilience and overall well-being in carers. This study evaluates the effectiveness of a self-directed e-coaching intervention for family carers of older adults aimed at reducing anxiety and depressive symptoms, while also assessing its impact on enhancing their resilience and overall well-being, particularly for those with mild levels of carer needs.

**Objective:**

This study aims to evaluate the effectiveness of a self-directed e-coaching intervention in reducing symptoms of anxiety and depression among carers of older adults using the 7-Item Generalized Anxiety Disorder Scale (GAD-7) and 9-Item Patient Health Questionnaire (PHQ-9), respectively, and also to examine secondary outcomes to determine its broader impact, including improvement in the level of carer needs, quality of life, caregiving burden, self-care efficacy, and resilience.

**Methods:**

This is a 3-arm randomized controlled trial that involves family carers of older adults living in Hong Kong, who will be randomly assigned to one of the e-coaching intervention groups or the control group through an online platform. The e-coaching intervention will consist of structured modules for self-directed learning on lifestyle intervention, family relationships, and emotion regulation. Participants in the intervention groups will either receive full content or partial content tailored to their caregiving needs. A total of 240 participants will complete the GAD-7 and PHQ-9 assessments at baseline, postintervention, and at a 3-month follow-up to evaluate changes in anxiety and depression scores. Secondary outcome measures will include standardized measurements assessing the level of carer needs, quality of life, caregiving burden, self-care efficacy, and resilience. It is hypothesized that participants in both e-coaching intervention groups will demonstrate a statistically significant reduction in anxiety and depressive symptoms at both postintervention and 3-month follow-ups compared to the control group, in terms of reductions in GAD-7 and PHQ-9 scores.

**Results:**

This randomized trial was funded for an original project period from January 2023 to January 2028. The enrollment commenced in April 2025 and is ongoing, with the expectation of closing enrollment in May 2026. We anticipate that data analyses will be completed by September 2026.

**Conclusions:**

The anticipated findings from this study could provide valuable insights into the potential of e-coaching as an accessible self-help intervention for improving mental health outcomes among carers of older adults, particularly those classified with mild levels of carer needs. If successful, this self-directed approach may offer a scalable solution to alleviate psychological distress and enhance overall well-being and resilience in this vulnerable population. The results will inform future mental health strategies and interventions tailored for carers, ultimately fostering a healthier caregiving environment.

## Introduction

### Background 

Population aging is a ubiquitous global phenomenon, with an estimated one-sixth of the world’s population expected to be aged 60 years or more by 2030 [1]. Hong Kong has become a significantly aged society, with 23.3% of its population aged over 65 years [2] as of 2024, and it is experiencing rapid aging at an accelerating rate. It is projected that by 2046, the percentage of the population aged over 65 years will increase to 36% [3]. By 2050, the estimated percentage of the global aging population is projected to be just 22% [1]. Consequently, Hong Kong is trending toward having one of the oldest populations in the world. The societal challenges that arise from a super-aged society will be significant if not addressed promptly.

In light of Hong Kong’s transition to a super-aged society, the role of family carers has become increasingly vital. There are approximately 280,000 family carers of older adults in Hong Kong [4], which accounts for about 3.79% of the local population. This percentage is notably lower than those in several European countries, such as 13% in Spain and Portugal and 25% in Denmark and Belgium [5]. Traditional Chinese cultural values may help explain this discrepancy, as strong family ties often lead carers to not identify themselves as carers [6,7]. Consequently, this lack of recognition can result in many family carers embarking on their caring journey without formal training, which frequently leads to elevated stress levels and associated emotional symptoms [8-10]. As a result, the burden on family carers in Hong Kong is considerable. Therefore, providing support for these family carers is essential to maintain an effective caregiving ecosystem for older adults aging in place in Hong Kong [11]. For instance, an intervention designed for carers of dementia patients in the United States has been adopted and tested in Hong Kong, yielding favorable results that demonstrate its effectiveness [12].

Longitudinal studies have documented the negative impact of informal caregiving on the mental health of family carers, which deteriorates as they enter the caregiving journey and as care demands increase [13,14]. Additionally, decreases in leisure time and sleep time are thought to negatively affect carers’ subjective well-being [15]. A cross-sectional study by Lee et al [16] found that 28% of carers of patients with Parkinson disease were diagnosed with at least one psychiatric disorder, including depressive disorder (11.1%) and insomnia (6.4%). These rates are significantly higher than those observed in the general population (depression: 2%‐5%; insomnia: 4%) [17].

Various aspects and risk factors affecting carers’ mental health have been explored. Poor mental health has been linked to caregiving burden and challenges faced by carers of older adults. A study involving Chinese family carers of older adults in Shanghai revealed that carers’ competence had a significant impact on their quality of life and was mediated by the caregiving burden [18]. The researchers emphasized the importance of early identification and targeted measures, such as reducing caregiving burden, to mitigate potential health-related issues among family carers. Additionally, a group of researchers led by Ho et al [19] conducted in-depth interviews with Chinese-Canadian women who were carers for their spouses with Alzheimer disease. They reported feeling obligated to ensure their spouses’ well-being and regarded institutionalization as highly undesirable. Many expressed feelings of fatigue and an inability to relax psychologically.

In Hong Kong, Chow and Ho [11] investigated depression among older spousal carers and found that depression in this group was largely influenced by individual psychological resources, such as purpose in life, caregiver burden, and personal well-being. They urged the development of mental health programs aimed at enhancing these psychological resources for spousal carers. Given the high prevalence of psychological disturbances among carers and the need to bolster their individual psychological resources, there is a pressing need for evidence-based interventions and effective service delivery models specifically designed for carers.

### Interventions for Carers

#### Family Relationships

Family relationships have been reported as one of the stressors for carers of older adults [20-22]. Several empirical studies have investigated interventions aimed at positively influencing family relationships through communication skills training in various forms, including peer facilitation [23], home visits [24], and internet programs [25]. In particular, a study by Drossel et al [26] explored the effect of dialectical behavior therapy (DBT) skills training for family carers of people with dementia in a group setting. DBT focuses on helping individuals regulate their emotions, improve interpersonal effectiveness, develop distress tolerance, and practice mindfulness and has been studied for its efficacy in treating mood disorders, such as depression and anxiety [27-30]. The results suggested that DBT skills training improved carers’ problem-focused coping, enhanced emotional well-being, and reduced sense of fatigue, although the isolated effects of each individual skill were not reported in the study. Therefore, further exploration of the impact of interpersonal effectiveness on carers’ family relationships and psychological well-being is essential.

#### Lifestyle Intervention

In recent decades, researchers have shown a growing interest in the connection between lifestyle choices and mental health. When implemented effectively, improvements in lifestyle can serve as a low-cost, self-sustaining intervention to promote mental well-being. In New Zealand, White et al [31] followed the diets of 281 young adults over 21 consecutive days and showed that eating fruits and vegetables led to positive effects. They found that the benefits of eating fruits and vegetables not only improved mood on the day of consumption but also extended to the following day. Studies in the United States and Europe found a similar connection between healthy diet, physical activities, and mental well-being [32,33]. Regarding the prevention of mood disorders, supportive evidence for lifestyle interventions has also been found. In one systematic review, Mammen and Faulkner [34] analyzed the results from 30 longitudinal studies and found that physical activity was negatively associated with the risk of subsequent depression, and low levels of physical activity (<150 minutes of walking per week) were effective in preventing depression. A meta-analysis [35] of physical activity interventions found small-to-medium effects (*g*=0.37) on overall mental health (burden, depression, stress, and quality of life), with the most notable effect on quality of life (*g*=0.74). Carers of older adults often report high stress levels due to the demanding and continuous nature of their caregiving responsibilities. It has been suggested that lifestyle interventions focusing on better nutrition, daily exercise, and mind-body practices can effectively improve the ability to manage stress [36].

#### Low-Intensity Cognitive Behavioral Therapy

Given the high prevalence of psychological distress among carers, there is a strong need to develop evidence-based interventions to alleviate their psychological symptoms and support them in dealing with the demands of caregiving. In the literature, low-intensity cognitive behavioral therapy (LiCBT) has been a primary focus of studies concerning psychological interventions for carers [37-39]. LiCBT is an approach to disseminate CBT that aims to improve the accessibility of psychological treatment in the United Kingdom [40]. Unlike traditional CBT, which typically involves longer sessions led by trained clinical psychologists, LiCBT is delivered in 30-minute weekly sessions over a span of 6 to 8 weeks by mental health workers. To improve the efficiency and accessibility of LiCBT, self-help materials, computerized programs, and group delivery formats are often used.

A meta-analysis by Kaddour et al [38] analyzed studies of LiCBT in addressing anxiety, depression, subjective burden, and distress among carers of people with dementia. They found that LiCBT had a significant effect in reducing psychological difficulties experienced by carers compared to nonactive control groups. Small effect sizes were found for anxiety (*g*=0.35), depression (*g*=0.27), and distress (*g*=0.33). A medium effect was found for caregiving burden (*g*=0.53). A meta-analysis [41] on the effectiveness of clinician-guided and self-guided online cognitive behavioral therapy in reducing anxiety symptoms found that there was no significant difference for follow-up effect, with only a very small effect size at posttreatment (*g*=0.16). These studies suggest that an online-based self-guided LiCBT is a potentially effective, cost-effective, and accessible intervention to alleviate the psychological difficulties of carers in the community.

#### Self-Directed e-Coaching Intervention

The studies mentioned above highlight the need to promote mental well-being and alleviate psychological disturbances in carers, emphasizing the effectiveness of family relationships, lifestyle, and LiCBT interventions. However, attending therapy groups in person may not be feasible for carers of older adults due to limited mobility or the challenge of leaving older adults alone at home. Carers often face barriers such as a lack of transportation, the absence of a secondary carer, and inflexible schedules that hinder participation in demanding interventions [42]. Programs conducted in community settings typically have lower attendance rates compared to home-based options, such as telephone counseling and technology-based interventions [42].

In this context, internet-based interventions offer a convenient and cost-effective alternative [43-45]. Online educational programs provide flexibility for family carers, while e-coaching merges technology with personalized support for behavior change [46,47]. Our intervention focuses on individuals with lower psychological risk who are comfortable using computer and mobile devices for internet connection. A series of online self-help modules, developed by clinical psychologists of the research team, synthesizes three key areas: (1) lifestyle medicine, (2) LiCBT, and (3) interpersonal effectiveness. The goal of the e-coaching is to alleviate stress and prevent early psychological symptoms from progressing into serious illness, using a self-directed approach to enhance resilience and quality of life among carers.

### Objectives

This study aims to evaluate the effectiveness of a self-directed e-coaching intervention in reducing symptoms of anxiety and depression among carers of older adults using the 7-Item Generalized Anxiety Disorder Scale (GAD-7) and 9-Item Patient Health Questionnaire (PHQ-9), respectively, and also for secondary outcomes, including multidimensional needs assessment, resilience, quality of life, caregiving burden, and self-care efficacy.

### Hypotheses

It is hypothesized that participants in both intervention groups, either receiving full content or partial content tailored to their caregiving needs, will show a greater reduction in anxiety scores and depression scores, as measured by the GAD-7 and the PHQ-9, respectively, at both the postintervention and 3-month follow-ups compared to those in the control group. It is also hypothesized that participants in both intervention groups will experience improvements in secondary outcomes, including the multidimensional needs assessment, resilience, quality of life, caregiving burden, and self-care efficacy.

## Methods

### Study Design

This study is a 3-arm, single-blinded randomized controlled trial developed in accordance with the SPIRIT (Standard Protocol Items: Recommendations for Interventional Trials) checklist (Table 1). The research team will perform individual randomization using statistical software and assign the content through an e-platform to carers with mild needs. It will consist of a 6-week intervention period, with a screening process to be conducted prior to the intervention implementation. Statistical comparisons will be performed at baseline (T0), immediately postintervention (T1), and at a 3-month follow-up (T2) (Table 1). This study will be conducted from April 2025 to September 2026.

**Table 1.** Schedule of enrollment, intervention, and assessments.

	Study period					
	Enrollment	Allocation	Postallocation			
Timepoint	*−t*_1_	0	Baseline	Treatment	Immediately postintervention	3 months postintervention
Enrollment						
Initial eligibility screen	✓					
Informed consent	✓					
Allocation		✓				
Interventions						
e-coaching 1				✓		
e-coaching 2				✓		
Control				✓		
Assessments						
JCCSP^a^ carer multidimensional need assessment	✓		✓		✓	✓
PHQ-9^b^			✓		✓	✓
GAD-7^c^			✓		✓	✓
EQ-5D-5L			✓		✓	✓
WHOQOL-BREF^d^			✓		✓	✓
ZBI-12^e^			✓		✓	✓
SCSES-10^f^			✓		✓	✓
CD-RISC-10^g^			✓		✓	✓

a JCCSP: Jockey Club Carer Space Project.

b PHQ-9: 9-Item Patient Health Questionnaire.

c GAD-7: 7-Item General Anxiety Disorder Questionnaire.

d WHOQOL-BREF: World Health Organization Quality of Life–BREF.

e ZBI-12: Zarit Burden Interview.

f SCSES-10: 10-Item Self-Care Self-Efficacy Scale.

g CD-RISC-10: 10-Item Conner-Davidson Resilience Scale.

### Ethical Considerations

The protocol for this study has been registered with ClinicalTrials.gov under the identifier NCT06604481 on September 18, 2024. The registration was completed prospectively before the enrollment of the first participant. We obtained ethical approval from the Human Research Ethics Committee at the University of Hong Kong (#EA240545). Informed consent will be obtained from all participants included in the study. To safeguard confidentiality, participant identities will be anonymized within the system. Withdrawal from the study is voluntary at any time and will not impact their access to other services offered by the project.

### Participant Recruitment

Potential participants will be recruited from 10 district-based carer support units and 8 shared venues operated by the 6 community partners. Recruitment will commence in April 2025. Given the challenges of identifying and engaging the target participants, a rolling recruitment strategy will be used until the group assignment is completed, which is anticipated to take 12 months. Carers will be recruited if they meet the inclusion criteria (Textbox 1), pass at least one threshold in the 2 specified parameters, and express a willingness to join.

Textbox 1. **Inclusion and exclusion criteria.**

Inclusion criteria

- Being an adult aged 18 years or above with a family member aged 60 years or above requiring caregiving
- Reporting a mild level of need in the Carer Multidimensional Need Assessment
- Providing at least 6 hours of care per week
- Self-identifying as a family carer
- Living in Hong Kong
- Able to read Chinese
- Voluntary participation
- Able to use an electronic device independently

**Exclusion criteria**

- Experiencing acute health conditions that hinder caregiving support or intervention participation
- Having been diagnosed with Alzheimer disease or other types of dementia

The screening process involves carers completing a standardized form comprising demographics items and their willingness to participate. They can fill this out independently or with assistance from project staff upon enrolling. The research team developed a 22-Item Carer Multidimensional Need Assessment, which involves 5 domains of needs including physical health, mental health, social support, caregiving needs, and caregiving ability. Carers’ needs are categorized into 4 distinct levels: low, mild, moderate, and high [48].

### Sample Size Estimation

Sample size was calculated using analysis of covariance adjusting for baseline, and the longitudinal linear mixed model will be used as the primary analysis to use all repeated measures and available data.

According to a prior meta-analysis, internet-based self-help interventions for mental health demonstrate a medium effect size when compared to passive control conditions such as care-as-usual and waitlist [49]. According to another systematic review and meta-analysis of the effectiveness of internet-based psychoeducation programs for carers of people with dementia, internet-based psychoeducation programs had a significant impact on reducing carers’ depressive symptoms, with an effect size of −0.19 compared to control conditions [50].

Power analysis was conducted with G*Power 3.1.9.7. Assuming a medium effect size in the analysis of covariance, *α*=.05, power=0.80 [51], a total of 158 participants are required to join the study. Considering a possible attrition rate of 33%, the research team will set the minimum sample size at 240, with 80 participants in each arm [52].

### Registration and Research Consent

Eligible participants will complete a structured registration and consent process prior to engaging with the e-coaching platform. A Qualtrics survey will be administered to collect demographic data (eg, age, gender), secure informed consent, and capture email addresses necessary for platform registration. After completing the survey, participants will be registered on the e-coaching platform using the provided email addresses, receiving a one-time login password along with detailed access instructions. Participation in tests, surveys, and interviews will not affect the quality of care they receive from the community. This study does not involve drug usage or medical treatment, and there are no anticipated physical or medical risks to participants.

### Randomization

Randomization will be conducted using random permuted blocks of three through an online computer randomization system. Participants will be randomly assigned to one of three groups: (1) the full intervention group, where participants will receive the complete e-coaching with full content (ie, 6 sessions); (2) the need-specific intervention group, where participants will receive tailored e-coaching sessions extracted from the full module (ranging from 2 to 6 sessions) based on individual needs; or (3) the control group, where participants will receive video-based educational information about caregiving for 6 weeks.

To maintain allocation concealment, the randomization sequence will be generated prior to participant recruitment and will be securely stored by the research team leader. This approach ensures that those enrolling participants cannot foresee group assignments, thereby minimizing selection bias. In terms of implementation, the intervention will be delivered through an e-platform accessible to participants. This platform will facilitate the distribution of content and support participant engagement throughout the study period. Outcome assessors will be blinded to group assignments to minimize the potential bias in outcome measurement. Participants will be informed that they may not know which group they are assigned to, and they will be encouraged to focus on their personal experiences with the content provided.

### Outcome Measures

The same set of outcome measures will be assessed in 3 groups and across different time points. An intention-to-treat principle will be used for data analysis, in which all participants will be assessed with the same set of outcome measures across 3 measurements, regardless of adherence to their assigned intervention. Several measures will be used to capture the between-group treatment effects, including the PHQ-9 and the GAD-7 as the primary outcomes. The 22-Item Carer Multidimensional Need Assessment, EQ5D5L, World Health Organization Quality of Life–BREF (WHOQOL-BREF), Cantonese short version of Zarit Burden Interview (CZBI-Short-12), 10-Item Self-Care Self-Efficacy Scale (SCSES-10), and 10-Item Connor-Davidson Resilience Scale (CD-RISC-10) will be captured as secondary outcomes. The survey will primarily be self-administered using Qualtrics, and we estimate that it will take approximately 1 hour for participants to complete. However, if participants are unable to finish the questionnaire on their own, we will facilitate their completion of the survey via face-to-face interviews at their choice of venue or via telephone or online interviews depending on their preference.

### Intervention

#### e-Coaching Intervention (1 and 2)

The experimental group receives an e-coaching intervention based on LiCBT, lifestyle intervention, and interpersonal effectiveness. This program is designed to operate on an e-platform individually in an interactive, story-based, and self-directed format. It consists of 6 weekly sessions, each lasting about 10 to 30 minutes. Intervention group adherence will be the number of assigned modules completed by the participants. In between each session, participants are assigned home practices to further reinforce the skills learned. e-Coaching 1 will include all content listed in Table 2. e-Coaching 2 will include content tailored to the specific needs of carers, which will be identified using the 22-Item Carer Multidimensional Need Assessment, covering 5 domains of needs, including physical health, mental health, social support, caregiving needs, and caregiving ability (Table 3). This approach ensures that each participant in e-coaching 2 receives more focused material that directly addresses their specific needs.

**Table 2.** Session plan of e-coaching intervention group.

Session	Content and activity	Home practice
Carer online self-directed platform	Introduction of the relationship between online self-directed content and the situation of carer Mindfulness practice	Nil
Lifestyle intervention	Healthy lifestyle Mindfulness practice (standing) Mindfulness practice (stretch)	Mindfulness practice Healthy lifestyle planning
Sleep	Myths about sleep Sleep cycles and hygiene Mindfulness and breathing relaxation practice	Mindfulness practice Breathing relaxation practice Sleep hygiene
Family relationships and communication	Unspoken rules in families Communication skills in the family Mindfulness practice	Mindfulness practice Identify unspoken rules in families Improving communication patterns with family members
Emotion regulation	Identify different emotions Understand emotion mechanism Regulate negative emotion	Mindfulness practice Accumulate positive emotions
Respite time for carers	What is respite service, and why does it matter? Planning respite Mindfulness practice	Mindfulness practice Planning respite time
Caregiving skills for the older adults	Educational content on information, resources, and skills of caregiving for older adults	Nil

**Table 3.** Domains of carer’ needs and e-Coaching modules assigned.

Needs or modules	Introduction	Emotion regulation	Caregiving skills	Sleep	Respite time for carers	Lifestyle intervention	Family relationships and communication
Mental health	✓	✓	✓	✓			
Physical health	✓			✓		✓	
Caregiving ability	✓	✓	✓		✓		
Social support	✓				✓		✓
Caregiving needs	✓	✓	✓				

#### Control Group

On the other hand, the placebo-controlled group attends 6 weekly online sessions that primarily provide video-based educational information in a self-directed format on the same e-platform. The focus of the content will be on practical caregiving skills and promoting general well-being, rather than targeting any particular psychological or mental health–related factors directly. The control group will include all content listed in Table 4. The total duration of the control group lasts about 50 minutes. Control group adherence will be the number of videos completed by the participants. The control condition is designed as an “information-only” educational comparator, reflecting standard educational resources typically accessible to caregivers in the community. It does not include the interactive e-coaching component or structured home practice and does not overlap with the content provided in the intervention arm. This approach allows us to test the intervention materials and evaluate the added value of interactive e-coaching and guided self-help practice over standard educational materials.

**Table 4.** Topics and content of control Group.

Topics	Content
Caregiving experience	Video regarding sharing of other carers’ experiences.
Introduction to knowledge of aging-related health issue and pain management	Videos of early detection of dementia and emotional problems. Videos address strategies for managing and alleviating chronic pain in older adults.
Caregiving resources	Videos of community support resources, including hotline, respite, equipment rental services, useful mobile apps, and support network of community centers.

### Outcome Measures

#### Primary Outcomes

##### 7-Item Generalized Anxiety Disorder

The GAD-7 is a widely used screening tool that measures the severity of generalized anxiety disorder by assessing the frequency of anxiety symptoms experienced over the past 2 weeks. Respondents rate their experiences on a 4-point scale from “not at all” to “nearly every day,” providing a reliable assessment of anxiety severity [53].

##### 9-Item Patient Health Questionnaire

The PHQ-9 is a self-report instrument used to assess depression symptoms, consisting of 9 items that correspond to the diagnostic criteria for major depressive disorder. Participants will rate the frequency of symptoms experienced over the past 2 weeks, helping to determine the severity of depressive symptoms [54]. The last item serves as a screening question for suicide risk, requiring further assessment if answered affirmatively.

### Secondary Outcomes

#### 22-Item Carer Multidimensional Need Assessment

The 22-Item Carer Multidimensional Need Assessment was developed based on a large-scale population survey on carers’ well-being in Hong Kong. This tool covers six need domains: (1) carers’ health challenges, (2) carers’ mental ill health, (3) lack of support, (4) high care challenges, (5) caregiving capacity, and (6) risk or need for immediate support.

#### EQ-5D-5L

The EQ-5D-5L is a standardized instrument for measuring general health status. It consists of 5 dimensions: mobility, self-care, usual activities, pain or discomfort, and anxiety or depression. For each dimension, respondents choose from 5 levels ranging from “no problems” to “extreme problems.” The EQ-5D-5L is a widely used, preference-based measure of health-related quality of life. It is designed for self-completion by respondents and is applicable to a wide range of health conditions and treatments [55].

#### The World Health Organization Quality of Life–BREF

The WHOQOL-BREF is a 28-item instrument developed by the World Health Organization to assess an individual’s quality of life across 4 domains: physical health, psychological health, social relationships, and environment. Each item is scored on a 5-point Likert scale, with higher scores indicating a better quality of life. The WHOQOL-BREF provides a concise yet comprehensive evaluation of an individual’s overall well-being, making it a widely used tool in research, clinical practice, and public health assessments. Policy 21 Hong Kong Limited has been commissioned by the Hong Kong Jockey Club Charities Trust to conduct a survey on the quality of life of the target service users in the Jockey Club’s elderly and elderly services. The survey results will help the Jockey Club to plan and formulate projects [56,57].

#### 12-Item Cantonese Short Version of the Zarit Burden Interview

The CZBI-Short-12 is a brief questionnaire used to measure carer burden in individuals caring for a family member with a chronic illness or disability. It has been validated in Hong Kong and a spoken Cantonese version of the 12-Item ZBI to assess the burden on carers in clinical and community settings. Each item subjectively measures caregiver burden on a 5-point Likert scale, with possible answers ranging from 0 (never) to 4 (very frequently) [58].

#### Self-Care Self-Efficacy Scale

The SCSES-10 is a measurement tool used to assess an individual’s self-efficacy. Self-care self-efficacy for chronic illness has been tested in the Hong Kong population. This 10-item instrument measures self-efficacy related to self-care maintenance, monitoring, and management in patients with chronic illness. Each item is rated on a 5-point Likert scale, ranging from 1 (not at all confident) to 5 (totally confident). A higher score represents a higher level of self-efficacy [59].

#### 10-Item Connor-Davidson Resilience Scale

The CD-RISC-10 is a 10-item, self-administered scale that assesses an individual’s level of resilience. Each item is rated on a 5-point Likert scale, ranging from 0 (not true at all) to 4 (true all of the time). The total possible scores range from 0 to 40, with higher scores being an indicator of high resilience [60,61].

### Statistical Analysis

#### Primary Outcome Analysis

The linear mixed model will be used to evaluate the hypothesis regarding whether there is a reduction in anxiety and depression symptoms in the intervention groups compared to the control group. The model will include treatment conditions (e-coaching 1, e-coaching 2, and Control), assessment time points (baseline, immediately post intervention, and 3 months post intervention), and their interaction (group-by-time) effects as fixed effects. To account for the correlation of repeated measures within individuals, a random intercept for participants will be included. Anxiety and depression scores will serve as the dependent variables in separate models. Statistical significance is defined as alpha levels less than .05. Planned contrasts will separately compare each active intervention group (e-coaching 1 and e-coaching 2) against the control group at immediately post intervention and 3-month follow-up to evaluate intervention efficacy. Holm-Bonferroni correction will be applied to control the familywise error rate across multiple contrasts.

#### Secondary Outcomes Analysis

Separate linear mixed models will be used to examine the effects of the e-coaching intervention on the secondary outcomes, which include the EQ-5D-5L, WHOQOL-BREF, the Cantonese Short Version of the Zarit Burden Interview, SCSES-10, and CD-RISC-10. The models will include treatment conditions (e-coaching 1, e-coaching 2, and Control), assessment time points (baseline, immediately post intervention, and 3 months post intervention), and their interaction (group-by-time) effects as fixed effects. Statistical significance will be defined as alpha levels less than .05.

For the 22-item Carer multidimensional assessment, a logistic mixed model will be used, as the outcome will be binary, with 0 indicating not mild need and 1 indicating mild need. The model will include treatment conditions (e-coaching 1, e-coaching 2, and control), assessment time points (baseline, immediately post intervention, and 3 months post intervention), and their interaction effects (group-by-time) as fixed effects. Statistical significance for this model will also be defined as alpha levels less than .05.

Item-level missing data will be minimized by Qualtrics’s function of validation requiring complete responses. Full information maximum likelihood will be used to use all available data and account for the incomplete data (eg, a participant missing follow-up).

### Sensitivity Analysis

Sensitivity analysis will be conducted using a model additionally adjusted for participants’ demographic characteristics such as sex and age or by restricting the sample to only active participants (eg, those with a number of modules completed greater than the median) to assess whether the findings are robust with alternative modeling and sample analyzed.

## Results

**Figure 1.**
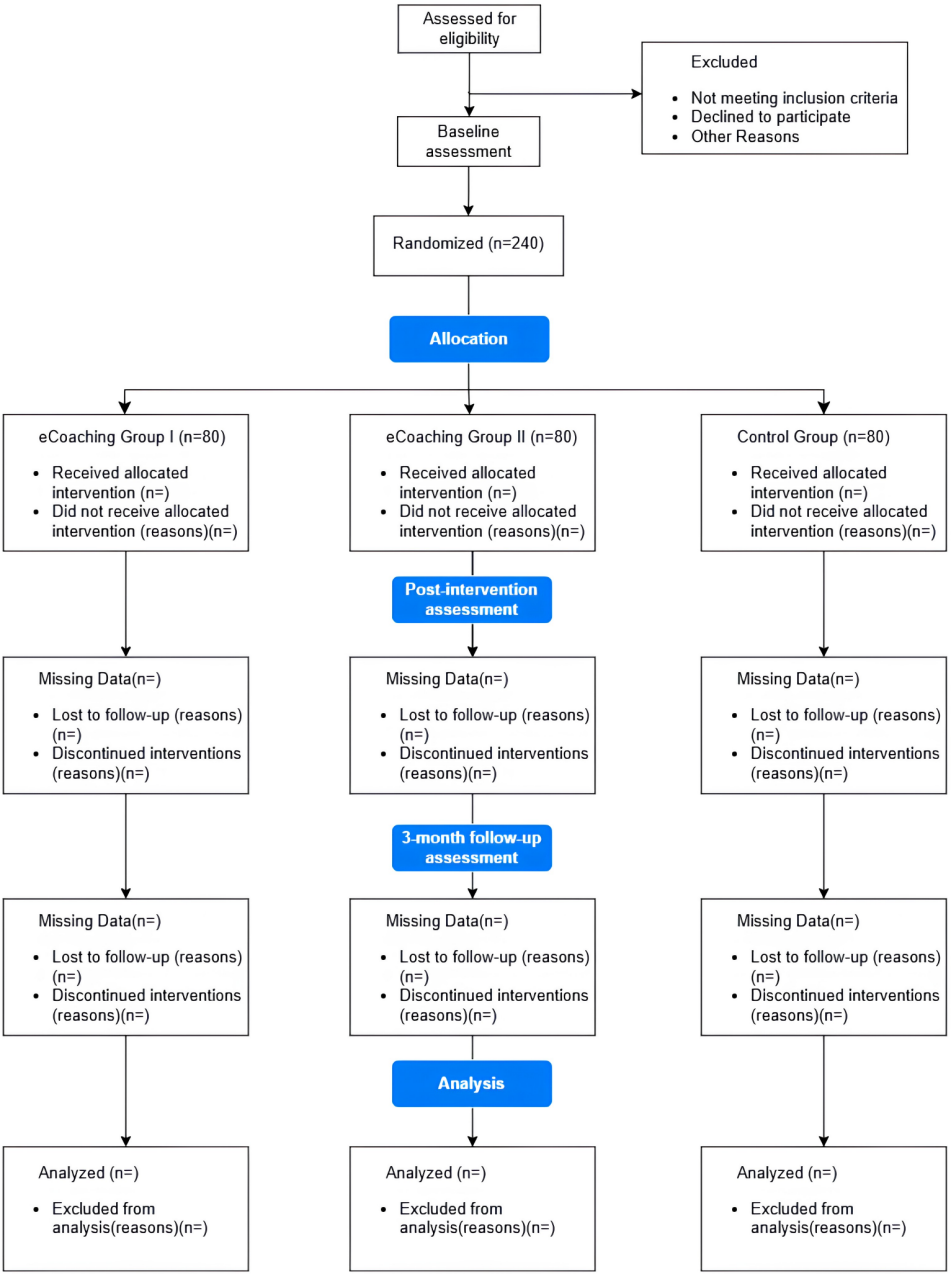
CONSORT (Consolidated Standards of Reporting Trials) flow diagram.

The CONSORT (Consolidated Standards of Reporting Trials) flow diagram is presented in Figure 1. This randomized trial was funded for an original project period from January 2023 to January 2028. The enrollment commenced in April 2025 and is ongoing with the expectation of closing the enrollment in May 2026. We anticipate that data analyses will be completed by September 2026.

## Discussion

### Principal Findings

The self-directed e-coaching intervention aims to support carers of older adults by addressing their mental health challenges. Existing literature indicates that carers often experience heightened levels of anxiety and depression, which can adversely affect their well-being and caregiving abilities. Our study aims to evaluate the effectiveness of this innovative intervention in reducing symptoms of anxiety, as measured by the GAD-7, and depression, as assessed by the PHQ-9. The hypotheses proposed that participants in the intervention group would demonstrate greater reductions in anxiety and depression scores compared to a control group, as well as improvements in secondary outcomes such as resilience, quality of life, and caregiving burden

### Summary of Findings

Preliminary insights suggest that self-directed e-coaching can be a viable alternative to traditional in-person interventions, addressing barriers that carers often face, such as limited mobility and inflexible schedules [43]. By offering a flexible online format, the intervention provides carers with the opportunity to engage with the material at their own pace, thereby enhancing accessibility and adherence. This aligns with previous findings that highlight the efficacy of internet-based interventions in improving mental well-being among carers [44,45].

The incorporation of LiCBT principles into the e-coaching model is particularly noteworthy. Meta-analyses have demonstrated LiCBT’s effectiveness in reducing psychological distress among carers, and our approach aims to leverage these techniques in a self-directed format [39].

### Strengths and Limitations

The use of a randomized controlled design enhances the rigor of our findings, allowing for robust comparisons between interventions and control groups. This methodological strength increases the reliability of our outcomes and strengthens the generalizability of the results. It can provide deeper insights into the mechanisms of change, although the reliance on self-report measures may introduce biases. Future research should consider incorporating more objective measures, such as behavioral assessments or physiological indicators, to complement self-reported data.

While the self-directed nature of the e-coaching intervention offers significant advantages, it may not suit all carers, particularly those with lower digital literacy or comfort with technology. Addressing these disparities through tailored support and guidance will be crucial for maximizing the intervention’s reach and effectiveness.

The control arm in this study is not dose-matched with the intervention arms in terms of the sessions’ length, which could introduce a limitation regarding attention-related effects. Future studies should therefore include a dose-matched control condition with similar sessions’ lengths to distinguish specific intervention effects from attention-related effects.

### Importance of This Study

This study is critical for advancing carer support, as it fills a significant gap in research on the effectiveness of self-directed interventions tailored for carers. By examining both primary outcomes—specifically anxiety and depression—and secondary outcomes such as resilience, quality of life, caregiving burden, and self-efficacy, we aim to conduct a thorough evaluation of the intervention’s overall impact. These findings will be instrumental in shaping future carer support programs, ensuring that interventions are designed to address the diverse and complex needs of this population.

Furthermore, the expected improvements in secondary outcomes will provide valuable insights into how the e-coaching intervention enhances overall carer well-being. This comprehensive evaluation not only contributes to a deeper understanding of the intervention’s effects but also aids in refining strategies that enhance relevance and effectiveness in clinical practice.

### Future Directions

As we move forward, further research is needed to explore the long-term effects of the e-coaching intervention and its potential mechanisms of change. Future studies should investigate how specific components of the intervention contribute to the observed improvements in mental health outcomes. Understanding these dynamics will be essential for optimizing the intervention and ensuring its sustainability. Moreover, it will be essential to refine the intervention based on participant feedback and outcomes, ensuring that it remains responsive to the evolving needs of carers.

### Conclusions

In conclusion, our study aims to provide valuable insights into the effectiveness of a self-directed e-coaching intervention for carers of older adults. By addressing both psychological symptoms and broader psychosocial factors, we hope to contribute to the growing body of knowledge that informs best practices in carer support and enhances the resilience and well-being of those in this critical role.

### Dissemination Plans

The trial findings will use multiple channels to reach professional and public audiences. The project findings will be disseminated through peer-reviewed journals and academic conferences to help inform efforts to promote community-based family carer support programs in Hong Kong.

## Abbreviations

CD-RISC-10: 10-Item Conner-Davidson Resilience Scale
CONSORT: Consolidated Standards of Reporting Trials
CZBI-Short-12: 12-Item Cantonese Short Version of the Zarit Burden Interview
DBT: dialectical behavior therapy
EQ-5D-5L: EuroQol-5 Dimensions-5 Levels
GAD-7: 7-Item General Anxiety Disorder Questionnaire
LiCBT: low-intensity cognitive behavioral therapy
PHQ-9: 9-Item Patient Health Questionnaire
SCSES-10: 10-Item Self-Care Self-Efficacy Scale
SPIRIT: Standard Protocol Items: Recommendations for Interventional Trials
WHOQOL-BREF: World Health Organization Quality of Life–BREF
